# URG4 overexpression is correlated with cervical cancer progression and poor prognosis in patients with early-stage cervical cancer

**DOI:** 10.1186/1471-2407-14-885

**Published:** 2014-11-26

**Authors:** Lan Zhang, He Huang, Longjuan Zhang, Teng Hou, Shu Wu, Qidan Huang, Libing Song, Jihong Liu

**Affiliations:** Sun Yat-sen University Cancer Centre, State Key Laboratory of Oncology in South China, Collaborative Innovation Centre for Cancer Medicine, Guangzhou, 510060 PR China; Department of Gynaecology Oncology, Sun Yat-Sen University Cancer Centre, Guangzhou, 510060 PR China; Laboratory of Surgery, First Affiliated Hospital, Sun Yat-Sen University, Guangzhou, 510080 PR China

**Keywords:** URG4, Cervical cancer, Prognosis, Concurrent chemotherapy and radiotherapy, Biomarker

## Abstract

**Background:**

Upregulator of cell proliferation 4 (URG4) has been implicated in the oncogenesis of certain cancers. However, the correlation between URG4 expression and clinicopathological significance in human cancer remains unclear. Therefore, this study investigated its expression and clinicopathological significance in cervical cancer patients.

**Methods:**

URG4 expression was examined using quantitative PCR (qPCR) and western blotting in normal cervical epithelial cells, cervical cancer cells, and eight matched pairs of cervical cancer tissues and adjacent noncancerous tissues from the same patient. In addition, immunohistochemistry (IHC) was used to examine URG4 expression in paraffin-embedded tissues from 167 cervical cancer patients (FIGO stages Ib1-IIa2). Statistical analyses were performed to evaluate associations between URG4 expression and prognostic and diagnostic factors.

**Results:**

URG4 was significantly upregulated in the cervical cancer cell lines and tissues compared with the normal cells and adjacent noncancerous cervical tissues. IHC revealed high URG4 expression in 59 out of the 167 (35.13%) cervical cancer specimens. Its expression was significantly correlated with clinical stage (*P* < 0.0001), tumour size (*P* = 0.012), T classification (*P* = 0.023), lymph node metastasis (*P* = 0.001) and vaginal involvement (*P* = 0.002). Patients with high URG4 expression, particularly those who received concurrent chemotherapy and radiotherapy (*P* < 0.0001), showed a shorter overall survival (OS) and disease-free survival (DFS) compared to those with the low expression of this protein. Multivariate analysis revealed that URG4 expression is an independent prognostic factor for cervical cancer patients.

**Conclusions:**

Our results demonstrated that elevated URG4 protein expression is associated with a poor outcome in patients with early-stage cervical cancer. URG4 may be a novel prognostic marker and therapeutic target for the treatment of cervical cancer.

**Electronic supplementary material:**

The online version of this article (doi:10.1186/1471-2407-14-885) contains supplementary material, which is available to authorized users.

## Background

Cervical cancer is the third most commonly diagnosed gynaecological cancer and the fourth leading cause of gynaecological cancer deaths worldwide, and it accounted for 9% (529,800) of the total new cancer cases and 8% (275,100) of the total cancer deaths among females in 2008. More than 85% of these cases and deaths occurred in developing countries, including China[1]. In China, there are approximately 130,000 new cases and 50,000 deaths due to cervical cancer per year[2]. Although the incidence and mortality of cervical cancer have shown downward trends, it is still the major cause of gynaecologic oncology-related death in developing countries, and it is a public health problem worldwide[1]. The treatment strategy for cervical cancer depends on the clinical stage, which is defined by the International Federation of Gynaecology and Obstetrics (FIGO) staging system. There are several traditional clinical variables that play important roles in the FIGO staging system and patient prognosis, including lymph node metastasis, tumour size and parametrial involvement[3, 4]. After surgery, patients with one or more of the abovementioned clinical variables require supplementary therapy. However, traditional pathological variables are not sufficiently reliable for predicting clinical outcomes or for guiding optimal treatment strategies. Many genes, such as annexin A2, Sam68 and HDAC10[5–7], have been reported to be potentially useful prognostic markers in cervical cancer, but there is still an urgent need for additional research to identify novel biomarkers to supply practical information for patient prognosis and suitable therapeutic options.

Upregulated gene 4 (URG4) has been identified as an oncogene with a full-length mRNA of 3.607 kb that encodes a protein of approximately 104 kDa in size. This gene may be associated with the onset of oncogenesis and cell cycle regulation[8–10]. URG4 was initially identified using subtractive hybridisation in hepatocellular carcinoma (HCC) cells[8]. Previous research on HCC and gastric cancer evaluating tissue culture and tumour formation in nude mice has demonstrated that URG4 promotes HepG2 and GES-1 cell growth and that it is associated with poor survival. In addition, elevated URG4 expression in HCC and gastric cancer cells leads to upregulation of cyclin D1, whereas low URG4 expression downregulates the expression of this gene. A study by Chan Xie has indicated that URG4 regulates cyclin D1 expression via the Akt/FOXO3 signalling pathway by mediating its proliferative effects on HCC cells[10]. In addition, some studies have shown that the URG4 expression is increased in different types of cancers[11–13]. However, the clinical significance of this gene in human cervical cancer remains unknown.

In the present study, we demonstrated that the expression of URG4 is upregulated in cervical cancer cells and surgical specimens. Moreover, its expression in cervical cancer is associated with clinical stage, tumour size, T classification, N classification and vaginal involvement. Multivariate analysis revealed that URG4 may be an independent biomarker for predicting cervical cancer prognosis. More importantly, its upregulation is indicative of a poor prognosis, particularly in patients receiving concurrent chemotherapy and radiotherapy. Our results suggest that URG4 may be an independent biomarker for prognosis and that it represents a therapeutic target for the treatment of cervical cancer.

## Methods

### Cell lines

A primary culture of normal cervical epithelial cells was established from a biopsy of non­cancerous cervical epithelium and was cultured in complete Keratinocyte­SFM medium (Invitrogen, Carlsbad, CA, USA). Patient consent was obtained prior to the use of the clinical materials for research purposes, and the patient consent and protocol were approved by Sun Yat-sen University Cancer Center Institutional Review Board. Eight human cervical cancer cell lines were purchased from the American Type Culture Collection (HeLa, HeLa 229, C-33A, MS751, SiHa and Ca Ski) and the Cell Bank of Type Culture Collection of the Chinese Academy of Sciences (HCC 94 and ME-180). The HeLa, HeLa 229, C-33A, MS751 and SiHa cells were cultured in Eagle’s minimum essential medium (Gibco BRL, Rockville, MD). The Ca Ski and HCC 94 cells were cultured in RPMI-1640 medium (Gibco BRL, Rockville, MD), and the ME-180 cells were cultured in McCoy’s 5A medium (Sigma) supplemented with 10% foetal bovine serum (FBS) (HyClone, Logan, UT, USA).

### Samples and clinical characteristics

This study was conducted on a total of 167 paraffin-embedded cervical cancer samples, which were histopathologically and clinically diagnosed at the Sun Yat-Sen University Cancer Centre between 1999 and 2005. The clinical and clinicopathological classifications and staging were determined according to the 2009 FIGO criteria. All of the patients enrolled in this study were only found to possess gynaecological tumour(s), and they were treated without preoperative radiotherapy, chemotherapy, or hormonal therapy. Patient consent was obtained prior to the use of the clinical materials for research purposes, and the patient consent and protocol were approved by Sun Yat-sen University Cancer Center Institutional Review Board. Clinical information pertaining to the samples is summarised in Table 1. The follow-up time for the primary cervical cancer cohort ranged from 0.8 to 187 months, and the median follow-up time was 64.06 months. The percentage of tumour purity in the sections adjacent to the tumours and the normal cervical tissues used for RNA extraction were estimated during routine histopathological analyses.

**Table 1:** **Clinicopathological characteristics and URG4 expression in the cervical cancer patients**

	Number of cases (%)
**Age, years**	
**≥42**	**93 (55.7)**
**<42**	**74 (44.3)**
**Squamous cell carcinoma antigen, ng/ml**	
**≤1.5**	**97 (58.1)**
**>1.5**	**51 (30.5)**
**No test**	**19 (11.4)**
**FIGO stage**	
**Ib1**	**68 (40.7)**
**Ib2**	**59 (35.3)**
**IIa1**	**38 (22.8)**
**IIa2**	**2 (1.2)**
**Tumour size, cm**	
**<4**	**106 (63.5)**
**≥4**	**61 (36.5)**
**Histological type**	
**Squamous cell carcinoma**	**153 (91.6)**
**Adeno cell carcinoma**	**14 (8.4)**
**Histological differentiation**	
**Well**	**8 (4.8)**
**Moderate**	**66 (39.5)**
**Poor**	**93 (55.7)**
**Deep stromal invasion**	
**No**	**67 (40.1)**
**Yes**	**100 (59.9)**
**Lymphovascular space involvement**	
**No**	**160 (95.8)**
**Yes**	**7 (4.2)**
**Positive parametrium**	
**No**	**162 (97)**
**Yes**	**5 (2.4)**
**Positive surgical margin**	
**No**	**164 (98.2)**
**Yes**	**3 (1.8)**
**Vaginal involvement**	
**No**	**157 (94.0)**
**Yes**	**10 (6.0)**
**T classification**	
**T1b1**	**113 (67.7)**
**T1b2**	**39 (23.4)**
**T2a**	**9 (5.4)**
**T2b**	**4 (2.4)**
**T4**	**2 (1.2)**
**N classification**	
**N0**	**140 (83.8)**
**N1**	**27 (16.2)**
**M classification**	
**No**	**160 (95.8)**
**Yes**	**7 (4.2)**
**Concurrent chemotherapy and radiotherapy**	
**No**	**59 (62.1)**
**Yes**	**36 (37.9)**
**Radiotherapy**	
**No**	**49 (68.1)**
**Yes**	**23 (31.9)**
**Recurrence**	
**No**	**148 (88.6)**
**Yes**	**19 (11.4)**
**Vital status (at follow-up)**	
**Alive**	**155 (92.8)**
**Dead**	**12 (7.2)**
**URG4 expression**	
**Low or no expression**	**108 (64.7)**
**High expression**	**59 (35.3)**
	**0)**

### qPCR

Total RNA samples from the cell lines and primary tumour materials were extracted using TRIzol reagent (Invitrogen, Carlsbad, CA, USA) according to the manufacturer’s instructions. The extracted RNA was pretreated with RNase-free DNase, and 2 μg of RNA was used for cDNA synthesis using random hexamers. The URG4 sense primer was 5′-CGCAATCATCTCCTTCCATT-3′, and the antisense primer was 5′-GATTTGGGAGAAGTAGCCCC-3′. For the glyceraldehyde-3-phosphate dehydrogenase (GAPDH) gene, the sense primer 5′-AATGAAGGGGTCATTGATGG-3′ and the antisense primer 5′-AAGGTGAAGGTCGGAGTCAA-3′ were used. The initial PCR was performed as follows: denaturation at 95°C for 10 min, followed by 44 cycles of denaturation at 95°C for 15 s, primer annealing at 60°C for 60 min, and a primer extension step at 65°C for 5 s. Upon the completion of these steps, a final extension at 95°C was performed before the reaction mixture was stored at 4°C. qPCR was then conducted to determine the fold increase of URG4 mRNA in each of the primary cervical tumours relative to paired adjacent noncancerous tissue taken from the same patient. The primers were designed using Primer Express v2.0 (Applied Biosystems). The expression data were normalised to the geometric mean of GAPDH expression to control for variability in expression levels, and all of the experiments were performed in triplicate.

### Western blotting

Cells at 70% to 80% confluence were washed twice with ice-cold phosphate-buffered saline (PBS) and lysed on ice in radioimmunoprecipitation assay buffer (RIPA; Cell

Signaling Technology, Danvers, MA) containing complete protease inhibitor cocktail (Roche Applied Sciences, Mannheim, Germany). Fresh tissue samples were ground to powder in liquid nitrogen and lysed using SDS-PAGE sample buffer. Equal concentrations of each protein sample (20 μg) were separated on 6% SDS polyacrylamide gels and transferred to polyvinylidene fluoride (PVDF) membranes (Immobilon P, Millipore, Bedford, MA). The membranes were blocked with 5% fat-free milk in Tris-buffered saline containing 0.1% Tween-20 (TBST) for 1 h at room temperature. The membranes were then incubated with anti-upregulator of cell proliferation 4 antibody (1:1000, Sigma, HPA020134) overnight at 4°C. URG4 expression was determined using a horseradish peroxidase-conjugated anti-rabbit IgG antibody (1:3000, Santa Cruz, SC-2004) and enhanced chemiluminescence (Pierce) according to the manufacturer’s protocols. The membranes were probed with anti-α-tubulin mouse monoclonal antibody (1:1000, Sigma, T5168) as a loading control.

### Immunohistochemical analysis

Immunohistochemical analysis was performed to assess alterations in protein expression in the 167 human cervical cancer tissues. Briefly, paraffin-embedded specimens were cut into 4-μm thick sections and baked at 65°C for 30 min. The sections were deparaffinised with xylenes and rehydrated. They were then submerged into EDTA antigenic retrieval buffer and microwaved for antigenic retrieval. Next, they were treated with 3% hydrogen peroxide in methanol to quench endogenous peroxidase activity, followed by incubation with 1% bovine serum albumin to block any nonspecific binding. The sections were then incubated with an anti-URG4 rabbit polyclonal antibody (1:150, Sigma, HPA020134) overnight at 4°C. Normal goat serum was used as a negative control. After washing with PBST, the tissue sections were incubated with a biotinylated anti-rabbit secondary antibody (Sigma), followed by further incubation with streptavidin-horseradish peroxidase complex (Sigma). The tissue sections were immersed in 3-amino-9-ethylcarbazole, counterstained with 10% Mayer’s haematoxylin, dehydrated and mounted in Crystal Mount.

The degree of immunostaining of the formalin-fixed, paraffin-embedded sections was evaluated independently by two observers who were blinded to the histopathological features of the samples and the patient data. The scores assigned by the two independent investigators were averaged, and they were based on both the proportion of positively stained tumour cells and the intensity of staining. The proportion of tumour cells was scored as follows: 1 (<10% positive tumour cells), 2 (10-50% positive tumour cells), 3 (50-75% positive tumour cells), and 4 (>75% positive tumour cells). Staining intensity was graded according to the following criteria: 0 (no staining); 1 (weak staining = light yellow), 2 (moderate staining = yellow brown), and 3 (strong staining = brown). The staining index was calculated as the product of the proportion of positive cells and the staining intensity score. Using this method of assessment, we evaluated URG4 expression in cervical cancer cells via a staining index (scored as 0, 1, 2, 3, 4, 6, 8, 9 or 12). The cut-off values for URG4 expression were chosen based on a measure of heterogeneity using the log-rank test with respect to overall survival (OS). The optimal cut-off values were assigned as follows: staining scores of ≥6 described tumours with high URG4 expression, and scores of ≤4 were assigned to those with low URG4 expression.

### Statistical analysis

The OS rate was the primary endpoint of this study, and the secondary endpoint was the disease-free survival (DFS) of the cervical cancer patients. OS was defined as the duration from the date of each patient’s hospitalisation to the date of death from any cause or to the censoring of the patient at the date of the last follow-up. DFS was defined as the time from hospitalisation to local, regional, or distant treatment failure, other second primary cancer, or death without evidence of a cervical or second primary cancer.

All statistical analyses were conducted using SPSS 11.0 statistical software. The relationships between URG4 expression and the clinicopathological characteristics were analysed using the chi-square test and Fisher’s exact test. Bivariate correlations between the study variables were calculated using Spearman’s rank correlation coefficients. Survival curves were plotted using the Kaplan-Meier method and were compared using the log-rank test. The clinicopathological characteristics, which are used extensively to predict prognosis in clinical practice, were evaluated using univariate and multivariate analysis with the forward Cox regression model. In all cases, a *P*-value of <0.05 was considered to be statistically significant.

## Results

### URG4 is overexpressed in cervical cancer cell lines

**Figure 1:**
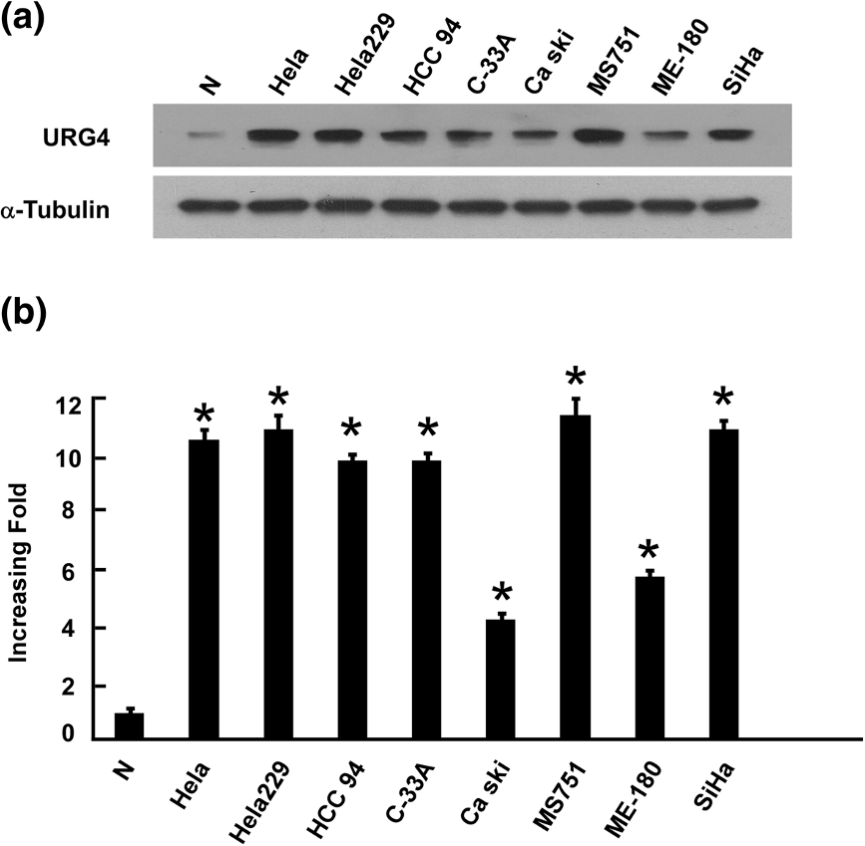
**Overexpression of URG4 mRNA and protein in cervical cancer cell lines.** **(a and b)** Expression levels of URG4 mRNA and protein in cervical cancer cell lines (HeLa, HeLa 229, HCC 94, C33a, Ca Ski, MS751, ME-180 and SiHa) and normal cervical cell lines were examined via western blotting **(a)** and qPCR **(b)**. The expression levels were normalised against α-tubulin and GAPDH, respectively. The error bars represent the standard deviation of the mean (SD), which was calculated from three parallel experiments. *P < 0.01.

To evaluate URG4 protein and mRNA expression in cervical cancer cell lines, we used western blotting and qPCR, and eight cervical cancer cell lines were assessed (HeLa, HeLa 229, HCC 94, C-33A, Ca Ski, MS751, ME-180 and SiHa) and compared with a normal cervical epithelial cell line (N). The URG4 protein was highly expressed in the cervical cancer cell lines and only weakly expressed in N (Figure 1a). URG4 mRNA expression was at least 4.5-fold greater in the cervical cancer cell lines compared to N (Figure 1b).

### URG4 is overexpressed in cervical cancer tissues

**Figure 2:**
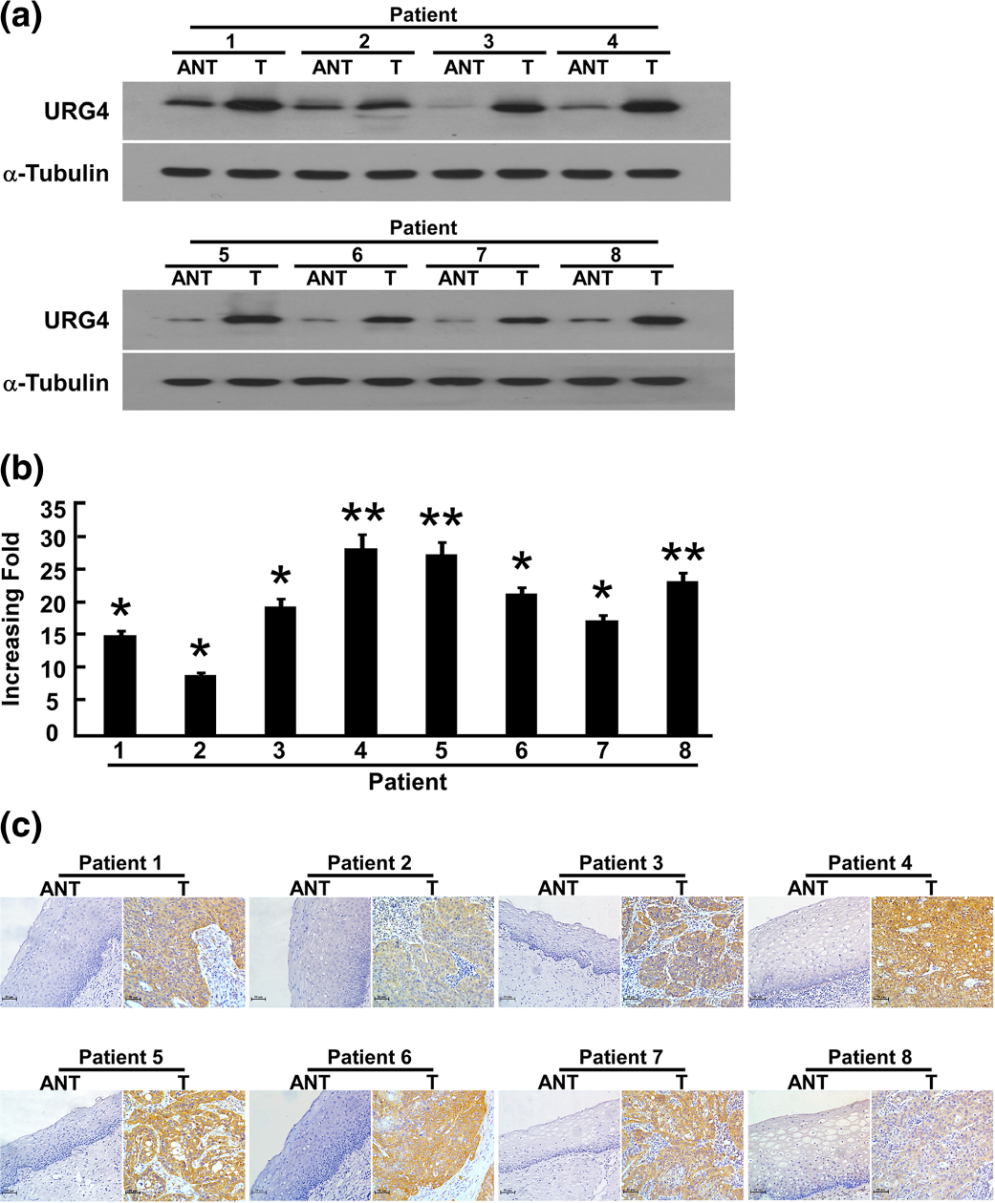
**Overexpression of URG4 mRNA and protein in cervical cancer tissues. (a)** Representative images of western blotting analyses of URG4 protein expression in eight matched pairs of cervical cancer tissues (T) and adjacent noncancerous tissues (ANT). α-Tubulin was used as the loading control. **(b)** The average T/ANT ratios of URG4 mRNA expression in the paired cervical cancer (T) and adjacent noncancerous tissues (ANT) were quantified using qPCR and normalised against GAPDH. The error bars represent the standard deviation of the mean (SD), which was calculated from three parallel experiments. **(c)** Immunohistochemical analysis of URG4 protein expression in eight pairs of matched cervical cancer tissues. *P < 0.05. **P < 0.01.

To determine whether URG4 is also highly expressed in human cervical cancer clinical samples, we performed qPCR and western blotting analyses on eight cervical tumour samples (T) that were matched with adjacent noncancerous tissue samples (ANT). As illustrated in Figure 2b, URG4 mRNA expression increased by 8.4- to 26.4-fold in all cervical cancer tissues compared to the matched adjacent noncancerous tissues. Consistent with these data, the URG4 protein was also upregulated in the cervical cancer tissues compared to the surrounding non-tumour regions (Figure 2b).

### URG4 overexpression is associated with clinical features of cervical cancer

**Figure 3:**
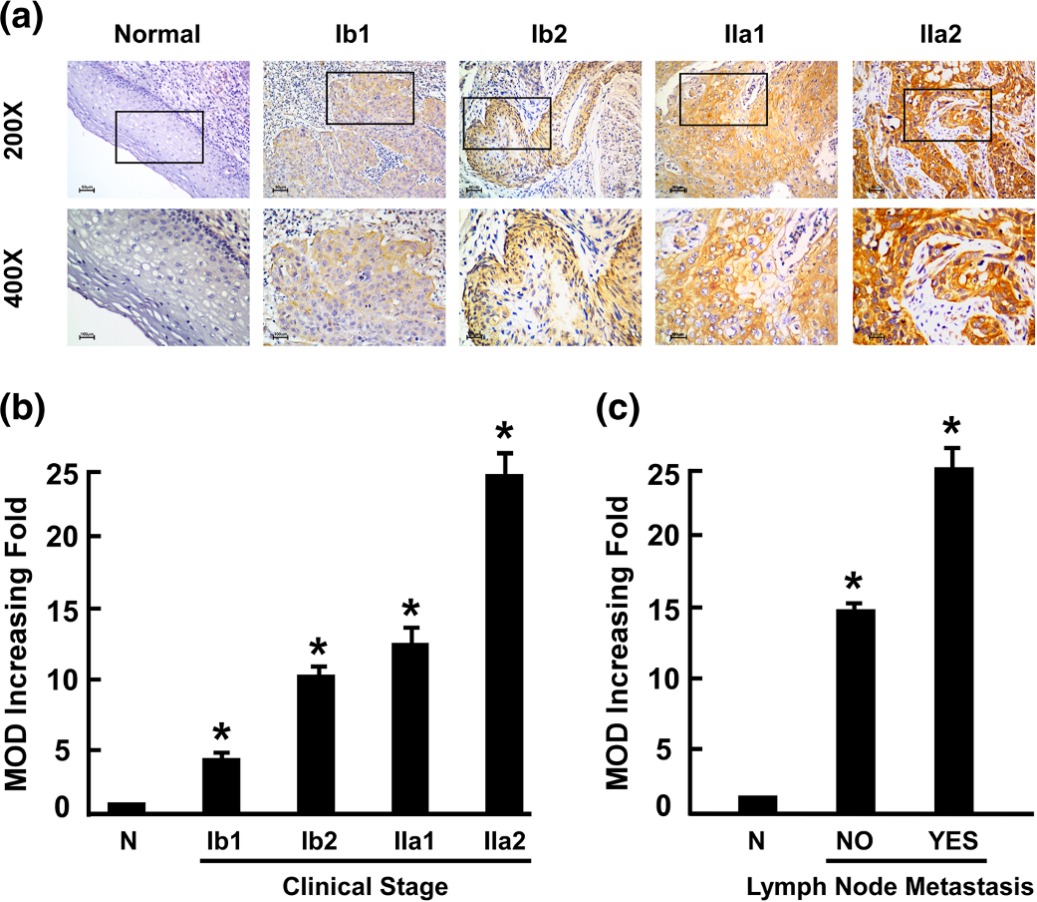
**Expression of the URG4 protein in cervical cancer tissues from patients at different clinical stages. (a)** Representative images from immunohistochemical analyses of URG4 expression in normal cervical tissues and cervical cancer tissues at different clinical stages. **(b)** The statistical analyses of the average mean optical density (MOD) of URG4 staining in normal cervical tissues and cervical cancer specimens at different clinical stages. **(c)** The statistical analyses of the average MOD of URG4 staining in the lymph node metastasis group and the lymph node metastasis-free group. *P < 0.05.

We investigated URG4 expression in 167 paraffin-embedded archived cervical cancer tissues using immunohistochemical staining. The samples included 68 stage Ib1 tumours, 59 stage Ib2 tumours, 38 stage IIa1 tumours and two stage IIa2 tumours. Among the 167 samples, high levels of URG4 protein expression were detected in 59 (35.5%), and weak or no staining was observed in the remaining 108 (64.7%, Table 1). The positive rate increased with increasing FIGO stage as follows: 16.2% for Ib1 (11/68), 45.8% for Ib2 (27/59), 50% for IIa1 (19/38) and 100% for IIa2 (2/2). URG4 was primarily localised to the plasma membrane (Figure 2). Furthermore, IHC staining showed that URG4 expression in the cervical cancer increased with increasing clinical stage (Figure 3a). Quantitative IHC analysis revealed that the mean optical density (MOD) values of URG4 staining in all of the cervical cancer samples were higher than those in the normal control cervical tissues. In addition, the MOD values of URG4 staining significantly increased with progression from stage Ib1 to IIa2 (*P <* 0.0001, Figure 3b). Taken together, these observations indicate that high levels of URG4 expression are associated with the clinical development of early-stage cervical cancer.

We further analysed the correlation between URG4 expression and the clinicopathological characteristics of the patients (Table 1). As summarised in Table 2, there were no significant correlations between URG4 protein expression and patient age, M classification, histological differentiation, SCC expression, histological type, deep stromal invasion, lymphovascular space involvement, positive parametrium or positive surgical margin in the patients with cervical cancer. However, URG4 expression was markedly associated with clinical stage (*P* < 0.001), T classification (*P* = 0.003), tumour size (*P* = 0.012), N classification (*P* = 0.001) and vaginal involvement (*P* = 0.004). These data were further confirmed by Spearman’s correlation analysis. As shown in Table 3, the correlations between URG4 expression and clinical stage, T classification, N classification and vaginal involvement were 0.327 (*P* < 0.0001), 0.250 (*P* = 0.001), 0.254 (*P* = 0.001) and 0.236 (*P* = 0.002), respectively. Moreover, the MOD values of URG4 staining were markedly higher in the lymph node metastasis group than in the lymph node metastasis-free group (*P* < 0.001, Figure 3c).

**Table 2:** **Correlation of clinicopathological characteristics and URG4 expression in cervical cancer patients**

Characteristics		Total (n = 167)	URG4		Chi-square test ***P***-value	Fisher’s exact test ***P***-value
			Low expression	High expression		
			(%)	(%)		
**Age (y)**	**≥42**	**93**	**56 (60.2)**	**37 (39.8)**	**0.798**	**0.873**
	**<42**	**74**	**46 (62.2)**	**28 (37.8)**		
**Clinical stage**	**Ib1**	**68**	**57 (83.8)**	**11 (16.2)**	**0.000**	**0.000**
	**Ib2**	**59**	**32 (54.2)**	**27 (45.8)**		
	**IIa1**	**38**	**19 (50.0)**	**19 (50.0)**		
	**IIa2**	**2**	**0 (0.0)**	**2 (100.0)**		
**T classification**	**T1b1**	**113**	**74 (65.5)**	**39 (34.5)**	**0.005**	**0.003**
	**T1b2**	**39**	**26 (66.7)**	**13 (33.3)**		
	**T2a**	**9**	**1 (11.1)**	**8 (88.9)**		
	**T2b**	**4**	**1 (25.0)**	**3 (75.0)**		
	**T4**	**2**	**0 (0.0)**	**2 (100.0)**		
**N classification**	**N**_**0**_	**140**	**91 (65.0)**	**49 (35.0)**	**0.001**	**0.002**
	**N**_**1**_	**27**	**11 (40.7)**	**16 (59.3)**		
**M classification**	**Yes**	**7**	**4 (57.1)**	**3 (42.9)**	**0.827**	**1.000**
	**No**	**160**	**98 (61.2)**	**62 (38.8)**		
**Histological differentiation**	**Well**	**8**	**7 (87.5)**	**1 (12.5)**	**0.058**	**0.069**
	**Moderate**	**66**	**34 (51.5)**	**32 (48.5)**		
	**Poor**	**93**	**61 (65.6)**	**32 (34.0)**		
**Expression of SCC**	**≤1.5**	**97**	**64 (66.0)**	**33 (34.0)**	**0.289**	**0.291**
	**>1.5**	**51**	**27 (52.9)**	**22 (47.1)**		
	**None**	**19**	**11 (57.9)**	**7 (42.1)**		
**Tumour size, cm**	**<4**	**106**	**76 (71.7)**	**30 (28.3)**	**0.012**	**0.018**
	**≥4**	**61**	**32 (52.5)**	**29 (47.5)**		
**Histological type**	**Squamous cell carcinoma**	**153**	**92 (60.1)**	**61 (39.9)**	**0.407**	**0.569**
	**Adeno cell carcinoma**	**14**	**10 (71.4)**	**4 (28.6)**		
**Deep stromal invasion**	**Yes**	**100**	**55 (55.0)**	**45 (45.0)**	**0.123**	**0.139**
	**No**	**67**	**47 (70.1)**	**20 (29.9)**		
**Lymphovascular space involvement**	**Yes**	**7**	**3 (42.9)**	**4 (57.1)**	**0.312**	**0.433**
	**No**	**160**	**99 (61.9)**	**61 (38.1)**		
**Positive parametrium**	**Yes**	**5**	**2 (40.0)**	**3 (60.0)**	**0.326**	**0.378**
	**No**	**162**	**100 (61.7)**	**62 (38.3)**		
**Vaginal involvement**	**Yes**	**10**	**1 (10.0)**	**9 (90.0)**	**0.002**	**0.004**
	**No**	**157**	**101 (64.3)**	**56 (35.7)**		
**Positive surgical margin**	**Yes**	**3**	**1 (33.3)**	**2 (66.7)**	**0.320**	**0.561**
	**No**	**164**	**101 (61.6)**	**63 (38.4)**

**Table 3:** **Spearman correlation analysis of URG4 versus clinicopathological factors**

Variables	URG4 expression level	
	Spearman correlation	***p***-Value
**Clinical staging**	**0.327**	**<0.000**
**T classification**	**0.250**	**0.001**
**N classification**	**0.254**	**0.001**
**Vaginal involvement**	**0.236**	**0.002**
**Tumour size**	**0.218**	**0.005**

Taken together, the expression of the URG4 protein is positively correlated with clinical stage, tumour size, T classification, N classification and vaginal involvement.

### Association between URG4 expression and patient survival

**Figure 4:**
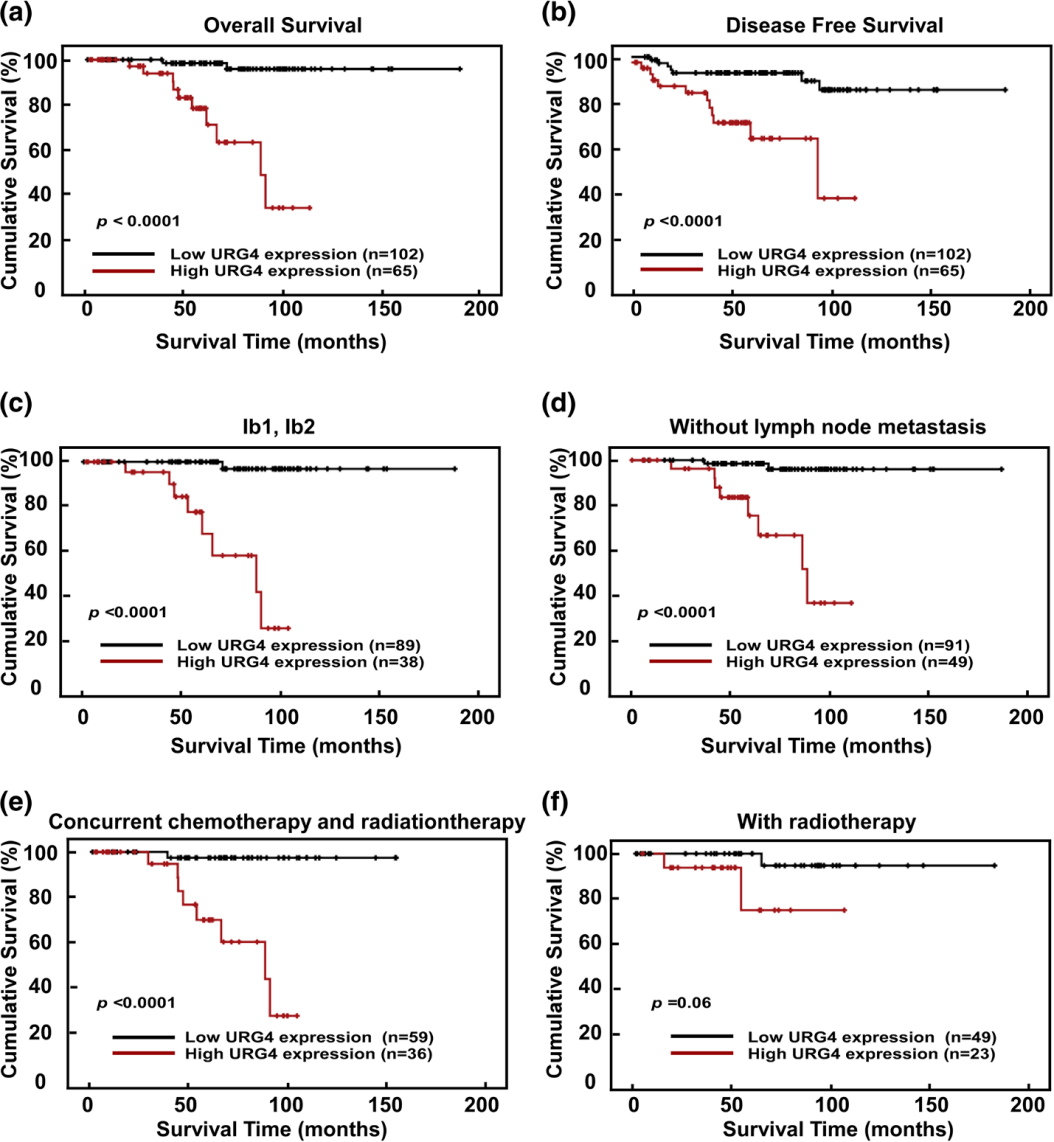
**Kaplan-Meier curves of univariate analysis data (log-rank test).** **(a and b)** The overall survival (OS) **(a)** and disease-free survival (DFS) **(b)** for the patients with high versus low URG4 expression. **(c)** The OS for the patients at stages Ib1-Ib2 with high versus low URG4 expression. **(d)** The OS for the patients without lymph node metastasis with high versus low URG4 expression. **(e)** The OS for the patients with high versus low URG4 expression who received concurrent chemotherapy and radiation therapy. **(f)** The OS for the patients with high versus low URG4 expression who received radiation therapy.

Patient survival analysis showed a clear negative correlation between URG4 protein expression and both the OS and DFS of cervical cancer patients (both *P* < 0.0001, Figure 4a, b). The cumulative OS and DFS rates for the patients with high levels of URG4 expression were 52.5% and 55.9%, respectively, whereas the rates were 97.1% and 89.8%, respectively, for the patients with low or no URG4 expression. Moreover, we analysed the prognostic value of URG4 expression in select patient subgroups that were stratified according to clinical stage, N classification, concurrent chemotherapy and radiotherapy and radiotherapy (RT). Patients with tumours exhibiting high URG4 expression had a significantly shorter OS compared to those with low-URG4-expressing tumours in the Ib1-Ib2 subgroup (log-rank test, *P* < 0.0001, Figure 4c), in those without lymph node metastasis (log-rank test, *P* < 0.0001, Figure 4d) and in those receiving concurrent chemotherapy and radiotherapy (log-rank test, *P* < 0.0001, Figure 4e). This was not the case for the IIa1-IIa2 subgroup (log-rank test, *P* = 0.485, Additional file1: Figure S1a), the lymph node metastasis group (log-rank test, *P* = 0.280, Additional file1: Figure S1b) or the radiotherapy group (log-rank test, *P* = 0.06, Figure 4f).

In addition, Cox regression analysis revealed that URG4 expression and deep stromal invasion were independent prognostic factors for poor OS in the cervical cancer patients (Table 4).

**Table 4:** **Univariate and multivariate analyses of various prognostic parameters in the patients with cervical cancer Cox regression analysis**

	Univariate analysis			Multivariate analysis*		
	No. patients	***p***	Regression coefficient (SE)	***P***	Relative risk	95% confidence interval
**URG4**			**2.755 (0.782)**	**<0.0001**		
Low expression	**108**	**<0.0001**			**Ref**	
High expression	**59**			**<0.0001**	**21.645**	**4.516-103.743**
**Deep stromal invasion**				**0.034**		
Yes	**100**	**0.264**			**Ref**	
No	**67**			**0.034**	**0.282**	**0.087-0.914**

*The factors in multivariate analysis included Age, Squamous cell carcinoma antigen, FIGO stage, Tumor size, Histological type, Histological differentiation, Deep stromal invasion, Lymphovascular space involvement, Positive parametrium, Positive surgical margin, Vaginal involvement, N classification, M classification, Concurrent chemotherapy and Radiotherapy, Radiotherapy and Expression of URG4.

(The endpoint is OS).

## Discussion

This is the first study to describe the association of URG4 upregulation with poor prognosis in early-stage cervical cancer patients. More importantly, high URG4 expression reduced the OS and DFS of these patients, particularly those who received concurrent chemotherapy and radiotherapy. Considering these findings, we suggest that URG4 is a potential novel marker for prognosis and represents a therapeutic target for the treatment of cervical cancer patients.

Clinical evidence has indicated that URG4 is elevated in patients with certain types of cancer. Its expression has been shown to be associated with tumour aggressiveness in osteosarcomas, prostate cancer and neuroblastomas[11–13]. These findings suggest that URG4 may play an important role in cancer development and progression. Therefore, we assessed whether it is also clinically associated with the development and progression of cervical cancer. We found that the URG4 mRNA and protein were highly expressed in cervical cancer cell lines and cervical cancer samples (Ib-IIa). We further analysed the relationships between URG4 expression and the clinical characteristics of patients with early-stage cervical cancer. There was a significant correlation between URG4 expression and clinical stage, T classification, tumour size, N classification and vaginal involvement, strongly supporting the hypothesis that this protein plays a role in the progression of cervical cancer and may represent a biomarker for the identification of subsets of cervical cancer patients with a more aggressive form of the disease. Univariate and multivariate analyses showed that high URG4 expression is a predictor of poor prognosis in these patients. Moreover, those with elevated expression showed a 52.5% cumulative OS rate, which was significantly lower than that in the patients with low expression levels (97.1%). Furthermore, the patients (Ib1-Ib2) with high URG4 expression levels had poor outcomes. These findings indicate that elevations in URG4 expression may be predictive of a poor prognosis and short survival time for early-stage cervical cancer patients.

Lymph node metastasis plays a very important role in the prognosis of cervical cancer patients. Recently, a large number of studies have identified certain genes associated with cervical lymph node metastasis, including Sam68, CRAM and EZH2[7, 14, 15]. Because all of patients were in early stage in our study, the number of patients with lymph node metastasis was small and they had a good prognosis. It was failed to show lymph node metastases was an independent prognostic factor for survival. We also showed that URG4 expression was high in the lymph node metastasis subgroup and that it was significantly correlated with lymph node metastasis. In a more detailed survival study, we observed a significant correlation between shorter OS and high URG4 expression in the “without lymph node metastasis” subgroup. This suggests that URG4 may be a useful prognostic marker for cervical cancer patients without lymph node metastasis. URG4 is regulated by the Akt-mediated phosphorylation of FOXO3a, which stimulates the cell cycle[8] or alters cyclin D1 levels by circumventing a cell cycle checkpoint (G1 to S phase)[9]. It is well known that Akt plays a role in tumour-induced lymphangiogenesis in colorectal carcinoma[16]. In the case of lymph node metastasis, we hypothesise that URG4 may regulate the expression of VEGF-C though the Akt signalling pathway by mediating lymphangiogenesis in cervical cancer cells. Further functional studies are needed to verify these findings to establish URG4 as a prognostic marker in cervical cancer and to clarify its role in carcinogenesis and progression.

For patients with lymph node metastasis, further treatments are required. To date, radical hysterectomy plus lymphadenectomy or chemoradiation have been the standard treatments for early-stage cervical cancer patients[17, 18]. A prospective, randomised clinical trial has shown that concurrent chemotherapy and radiotherapy improves the survival time of early-stage cervical cancer patients with high-risk prognostic factors compared with RT alone[19]. In our study, the patients with any of the “high-risk” prognostic factors, including pelvic lymph node metastasis, positive parametrial involvement, positive surgical margin, deep stromal invasion and large tumor size (over 4 cm), received chemotherapy and radiotherapy. The patients who were only with lymph vascular space invasion or vaginal involvement received RT. However, those who refused concurrent chemotherapy were treated with RT alone. We found that higher URG4 expression was correlated with a significantly shorter OS in the concurrent chemotherapy and radiotherapy subgroup. However, no correlation was observed in the subgroup receiving only radiotherapy. This indicates that URG4 expression is a more significant predictor of the prognosis of early-stage cervical cancer patients who require concurrent chemotherapy and radiotherapy.

Recently, a number of studies have focused on targeted drugs for the treatment of cervical cancer. Tewari *et al.* have found that chemotherapy combined with bevacizumab improves OS in recurrent, persistent or metastatic cervical cancer patients[20]. Nogueira *et al.* have revealed that EGFR inhibitors combined with chemoradiation result in high levels of complete remission in patients with locally advanced cervical cancer[21]. Consistent with the above findings, our study exploring URG4 as a target of cervical cancer has shown extremely promising results that may be of future clinical value.

## Conclusions

In conclusion, this is the first study to assess the expression and clinical significance of URG4 in early-stage cervical cancer. URG4 could represent a prognostic biomarker and therapeutic target for early-stage cervical cancer patients.

## Electronic supplementary material

Additional file 1: Figure S1: Kaplan-Meier curves with univariate analysis (log-rank test). **(a)** The OS for the patients with stages IIa1-IIa2 cervical cancer and high versus low URG4 expression. **(b)** The OS for the patients with lymph node metastasis and high versus low URG4 expression. (TIFF 269 KB)

## Abbreviations

URG4: Upregulator of cell proliferation 4
IHC: Immunohistochemistry
OS: Overall survival
DFS: Disease-free survival
FIGO: International federation of gynaecology and obstetrics
HCC: Hepatocellular carcinoma
PBS: Phosphate-buffered saline
RIPA: Radioimmunoprecipitation assay buffer
MOD: Mean optical density
EDTA: Ethylenediaminetetraacetic acid
PVDF: Polyvinylidene fluoride
RT: Radiotherapy
N: Normal cervical epithelial cell line.
