# Supplementary figures and images for: URG4 overexpression is correlated with cervical cancer progression and poor prognosis in patients with early-stage cervical cancer
Source: BMC Cancer. 2014 Nov 26;14:885. doi: 10.1186/1471-2407-14-885 (PMC4259088; doi:10.1186/1471-2407-14-885)

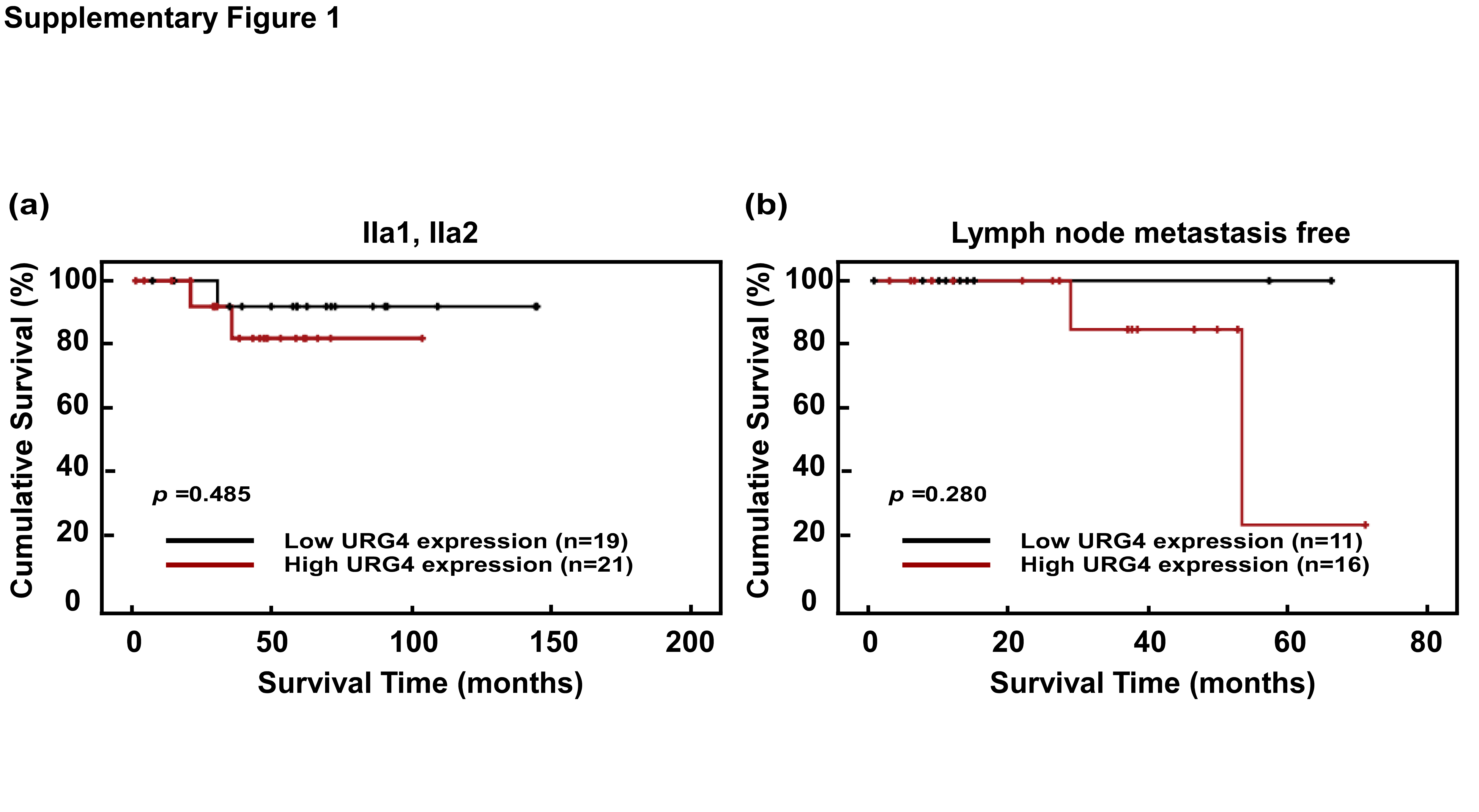

Supplement: Supplementary file 1 — Additional file 1: Figure S1: Kaplan-Meier curves with univariate analysis (log-rank test). (a) The OS for the patients with stages IIa1-IIa2 cervical cancer and high versus low URG4 expression. (b) The OS for the patients with lymph node metastasis and high versus low URG4 expression. (TIFF 269 KB) [file 12885_2014_5058_MOESM1_ESM.tiff]
